# Impact of a Digital Leakage Notification System on Healthcare Resource Utilisation and Costs for People with Stomas in the United Kingdom: Analysis of Interim Results from a Prospective Longitudinal Study

**DOI:** 10.3390/jmahp14020029

**Published:** 2026-05-06

**Authors:** Alexandra Baxter, Esben Bo Boisen, Majken Linnemann Jensen, Aamir Shaikh

**Affiliations:** 1 Coloplast A/S, 3050 Humlebæk, Denmark; dkebb@coloplast.com (E.B.B.);; 2 Coloplast Ltd., Peterborough PE2 6FX, UK

**Keywords:** stoma, leakage, digital leakage notification system, health-related quality of life, cost, healthcare resource utilisation

## Abstract

**Background/Objectives**: Leakage of stomal effluent adversely affects the quality of life for people living with a stoma and increases healthcare resource utilisation and costs. The Heylo™ digital leakage notification system (DLNS) detects early signs of leakage under the baseplate, enabling proactive stoma management. We evaluated the impact of the DLNS on stoma-related costs in a real-world UK setting. **Methods**: This costing analysis used data from the first 100 DLNS users with 6 months of follow-up in an ongoing longitudinal observational study (NCT06554015). Costs were calculated as 3-month totals before DLNS (baseline): Month 3, and Month 6 for stoma-related healthcare provider consultations, hospitalisations, and ostomy solution components (pouching systems, supporting products, and DLNS sensor layers). Least squares (LS) means were estimated using a mixed model. **Results**: Total LS mean costs for stoma-related consultations plus ostomy solution use decreased significantly from baseline by 21.9% at Month 3 (−£304 [95% CI: −£456, −£153]; *p* = 0.001) and 24.5% at Month 6 (−£340 [95% CI: −£504, −£175]; *p* < 0.001). Stoma-related consultation costs decreased by 50.7% at Month 3 (−£309 [95% CI: −£447, 171]; *p* < 0.001) and 57.9% at Month 6 (−£353 [95% CI: −£493, −£213]; *p* < 0.001), driven by fewer physician and stoma care nurse consultations. Mean ostomy solution costs remained similar from baseline (£784/person) to Month 6 (£782/person; *p* = 0.955) as DLNS sensor layer costs were offset by less use of other ostomy products. **Conclusions**: Initiation of the Heylo™ DLNS was associated with significant cost savings from reduced stoma-related consultations, pouching systems, and supporting product use.

## 1. Introduction

Leakage of faecal effluent remains one of the most challenging and distressing complications for people living with an intestinal stoma [1,2]. Even many years after surgery, individuals frequently report persistent leakage, worry about leakage, peristomal skin complications, and a substantial burden of day-to-day stoma care [3,4,5]. These issues have profound implications for quality of life (QoL), work productivity, and healthcare resource utilisation [6,7,8,9,10].

Stoma leakage under the baseplate is often undetectable by users and can precipitate a cycle of skin irritation, device failure, and worsening leakage [9,11,12,13]. Approximately 46% of people with stomas in the UK report experiencing leakages weekly, and 29% report leakages outside the baseplate (e.g., onto clothes, bedding; referred to here as “leakage” or “leakage events”) at least monthly [9]. More than 70% of people with stomas experience peristomal skin complications within a 6-month period, which negatively affect QoL and work productivity [4,10] and are associated with increased healthcare utilisation and costs [5]. Recurring leakage events drive increased stoma product use, nurse consultations, and persistent leakage-related distress, leading to higher costs for unresolved leakages [14]. Recent research has also highlighted broader consequences of leakage, including worry-driven behaviours such as premature pouch changes, overuse of supporting products, and withdrawal from work and social activities [7,9].

The Heylo™ (Coloplast A/S, Humlebæk, Denmark) digital leakage notification system (DLNS) is designed to detect and notify users when leakage spreads outside the baseplate, enabling patients to proactively manage it. Using Heylo™ has been shown to reduce leakage events, improve leakage-related QoL, and reduce unplanned pouching system changes [15,16,17]. An ongoing observational study (NCT06554015) is evaluating 12-month outcomes associated with Heylo™ use in real-world stoma care [18]. The interim results from the first 100 users showed a 51% reduction in leakage events, significant improvements across QoL domains, and reductions in time spent with healthcare professionals (HCPs) [18]. To complement the clinical and QoL evaluations, we performed a costing analysis to quantify changes in healthcare resource utilisation based on interim data from the first 100 participants with 6 months of follow-up.

## 2. Materials and Methods

This costing analysis was performed using data from an ongoing, prospective observational study (NCT06554015) in the United Kingdom. The design, methods, and interim results from the study have been reported by Vestergaard et al. (2026) [18]. Briefly, the longitudinal, single-arm study aims to follow 300 patients with stomas using the DLNS for 12 months. Eligible participants are adults (≥18 years) with an ileostomy or colostomy who initiated the DLNS (within 7 days of enrolment) and did not have a planned stoma reversal within 6 months. This study uses a pre/post design to compare patient-reported data before or within 7 days of initiating the DLNS (the “baseline” visit) with follow-up data captured in 3-month periods up to Month 12. This study is being conducted in accordance with the Declaration of Helsinki, ISO 14155:2020, [19] and European Medical Device Regulation 2017/745 [20]. According to the Health Research Authority, this study did not require review by a National Health Service (NHS) Research Ethics Committee. All participants provided informed consent.

Participants completed questionnaires providing demographic and clinical characteristics, leakage-related QoL, occurrence of leakages, use of ostomy products, the number and duration of stoma-related HCP visits and hospitalisations, and work productivity. Use of ostomy products includes the number of times the pouching system baseplate was changed (or entire system for one-piece users), use of supporting products (accessories), and how often the DLNS sensor layer was used during the previous 2 weeks.

This analysis focuses on the costs of stoma-related HCP consultations and hospital admissions, and the costs of the ostomy solution, which included the pouching system, supporting products, and DLNS sensor layers for the first 100 participants with follow-up data through Month 6.

The results of the planned Month 6 interim analysis provided the basis for the study population and healthcare resource utilisation data for this costing analysis [18]. The intent-to-treat population had a mean age of 50.9 years and was 61% female. Participants had been living with a stoma for an average of 7 years (70% for >1 year; 30% for ≤1 year), and most had an ileostomy (86%; 14% had a colostomy) [18]. Most participants used a 1-piece ostomy solution (81%) and a convex baseplate (72%). Participants reported using the DLNS with an average of 6.6 of the previous 10 baseplates at Month 3 (standard deviation [SD], 3.7; range, 0–10) and 5.6 of the previous 10 baseplates at Month 6 (SD, 4.1; range, 0–10). At Month 6, a 51% decrease (95% CI: 31%, 65%) was observed in the least squares (LS) mean number of leakage events during the previous 2 weeks, from an LS mean of 3.56 leakage events at baseline (95% CI: 3.02–4.19) to 1.76 events at Month 6 (95% CI: 1.25–2.47; *p* < 0.001) [18].

All variables of interest were costed for each participant at every study visit. All costs were reported as 3-month totals at each timepoint; for endpoints collected using shorter recall periods (e.g., 2 weeks), observed values were extrapolated to a 3-month period to estimate total costs. Baseline data were collected at DLNS initiation, with costs representing the period from 3 months before initiation up to the initiation date. Device costs were derived from the July 2025 Drug Tariff [21], and healthcare costs were sourced from the Personal Social Services Research Unit (PSSRU) Unit Costs of Health and Social Care 2024 [22] and the NHS National Cost Collection 2023/24 [23]. All costs were calculated in British Pound Sterling (£). Ostomy solution costs were calculated for pouching system changes, use of supporting products (excluding the DLNS), and use of DLNS sensor layers. Total ostomy solution costs were calculated as the sum of the pouching system costs, supporting product costs, and DLNS sensor layer costs for each participant at each visit. No imputation of missing data was performed if all three data points were missing.

Participants reported their pouching system (1-piece open, 1-piece closed, or 2-piece) and shape of baseplate (flat, convex, or concave), which was used to classify each system (e.g., 1-piece open flat, 1-piece open convex). Ostomy solution components were reported at baseline and at Months 3 and 6 to capture any changes in the types of products used. The cost of each product category was calculated using the weighted averages of pouching system market shares published by Boisen et al. (2025) [24] and was updated using the July 2025 Drug Tariff prices (Supplementary Materials Tables S1 and S2) [21].

Pouching system usage was collected using a 2-week recall period and extrapolated to estimate the 3-month total costs. Participants using 1-piece systems reported the number of pouching systems used; those using 2-piece systems reported the number of baseplates. Most participants used 1-piece systems, consistent with UK market distribution [25]. Usage values ranged from 0–30 (>30 was coded as 31). Missing data on the system type or baseplate shape were imputed using the lowest-cost product.

Costs of supporting products were based on the weighted averages of manufacturer market share splits reported by Boisen et al. (2025) [24] and were updated using the July 2025 Drug Tariff prices (Supplementary Materials Table S3) [21]. For belts, a weighted average of the most commonly used products from five manufacturers was applied (Supplementary Materials Table S4). As with the pouching systems, supporting product use was collected using a 2-week recall period, with usage between 0 and 30 (or >30, coded as 31), extrapolated to 3 months. For bottles, sprays, and belts, where reported quantities occasionally exceeded what would be considered reasonable prescribing patterns, usage was adjusted according to guidance based on the 2023 PrescQIPP UK prescription recommendations to ensure that supporting product costs were not overestimated [26]. For belts, maximum 3-month usage was capped at one belt, reflecting the guidance of 3 belts per year while allowing for additional need among people with complex stomas (Supplementary Materials Tables S3 and S4).

Participants reported how many times the DLNS was used out of their last 10 pouching system changes (or baseplate changes for 2-piece systems). This proportion was applied to each participant’s reported number of pouching system changes to estimate their sensor layer use, which was then multiplied by the unit cost of a DLNS sensor layer to calculate 3-month DLNS costs at each visit. The cost of the DLNS starter kit was excluded to account for current UK practice. Only the costs of reimbursed sensor layers were included.

Costs of stoma-related HCP consultations were estimated using the participant-reported number and average duration of stoma-related contacts based on a 3-month recall period. Participants could report between 0 and 15 visits or >15 visits, which was coded as 16 visits. Each consultation type was costed using either the NHS National Cost Collection 2023/2024 [23] or the PSSRU Unit Costs of Health and Social Care 2024 (Supplementary Materials Table S5) [22]. The reported number of contacts was directly matched to the relevant unit cost from the National Cost Collection, except for general practitioner (GP) visits, where the self-reported average duration of visits was costed using the PSSRU hourly patient-contact cost. Duration categories were <15 min, 15–30 min, 31–45 min, 46–60 min, 1–1.5 h, or user-entered number of hours; the upper bound of ranges was used to reflect the typical booked consultation time.

Costs of stoma-related hospital admissions were estimated using participant-reported numbers of admissions with an overnight stay in the previous 3 months. Participants could select between 0 and 30 admissions or choose >30, which was coded as 31. Since specific reasons for admission were not collected, an estimated average cost per admission was applied using secondary care data derived from the English Hospital Episode Statistics (HES) database produced by NHS England (©2025; all rights reserved) [27] and accessed via the Ostomy Care Interactive Dashboard (CorEvitas, LLC, Waltham, MA, USA). The Ostomy Care Interactive Dashboard is a bespoke dashboard providing national and local data on secondary care activity, including ICD-10 diagnosis codes, Office of Population Censuses and Surveys (OPCS) procedure codes, and Emergency Care Dataset (ECDS) entries. These data were reused with permission from NHS England under a data-sharing agreement (September 2025). HES data are released to the general public under strict guidelines that protect the privacy and confidentiality of patients [28]; only aggregated, anonymised data were used, and small-number suppression rules were applied in accordance with the NHS England guidelines. The 20 most commonly reported ICD-10 codes for people with colostomies and ileostomies between April 2023 and March 2025 were extracted (Supplementary Materials Table S6). Codes were evaluated for aetiological relevance to having a stoma, and the remaining codes were mapped to Healthcare Resource Group (HRG) codes using the HRG Code-to-Group workbook [29]. The total average cost of an admission (£2618.15) was calculated across elective inpatient, non-elective inpatient long-stay, and non-elective inpatient short-stay HRG activities (Supplementary Materials Table S7).

A sensitivity analysis assessed the impact of data-adjustment rules that limited the upper quantities of supporting products based on prescribing guidance. LS means and LS mean differences (baseline vs. Month 3 and Month 6) for pouching system costs, supporting product costs, and total ostomy solution costs were compared between the adjusted and unadjusted datasets to evaluate the validity of the adjustments.

An exploratory subgroup analysis was conducted to examine the impact of the time since stoma surgery on pouching system costs, supporting product costs, stoma-related consultation costs, and stoma-related hospital admission costs. Time since surgery was stratified as newly discharged patients (≤12 months post-surgery at baseline) and experienced users (>1 year since stoma surgery at baseline).

Category-level costs (pouching systems, supporting products, DLNS sensor layers, total ostomy solution costs, stoma-related consultations, stoma-related hospital admissions, and combined ostomy solution plus consultation cost) were analysed using LS means, standard errors (SE), and 95% confidence intervals (CIs) estimated for each visit using a mixed model with an unstructured covariance matrix to account for correlated repeated measurements. LS mean differences for Months 3 and 6 compared with baseline were also calculated. Sample medians and upper and lower quartiles were reported descriptively. Sample means, standard deviations (SD), medians, and quartiles were reported for individual supporting products, DLNS sensor layer use, and individual consultation types. In the subgroup analysis, LS means were used when available, and sample means were used otherwise. All analyses were conducted using SAS version 9.4 (SAS Institute Inc., Cary, NC, USA) and Microsoft Excel (Microsoft Corporation, Redmond, WA, USA).

All costs were valued in 2025 GBP and converted to EUR and USD using the average GBP–EUR and GBP–USD exchange rates for 15 September 2025, sourced from the Bank of England daily spot exchange rates against the GBP for the date that the costings analysis was performed (available at: https://www.bankofengland.co.uk/boeapps/database/Rates.asp?TD=15&TM=Sep&TY=2025&into=GBP&rateview=D); accessed on 10 February 2026. The price year and conversion approach are reported in line with CHEERS recommendations.

## 3. Results

### 3.1. Costs Associated with Stoma-Related HCP Consultations and Hospitalisations

Costs associated with stoma-related HCP consultations decreased significantly from baseline by 50.7% at Month 3 (−£309 [95% CI: −£447, −£171]; *p* < 0.001) and by 57.9% at Month 6 (−£353 [95% CI: −£493, −£213]; *p* < 0.001) (Table 1). The most notable decreases were observed for consultations with stoma nurses in the clinic (–76.6% at Month 6) and non-face-to-face nurse consultations (–68.7% at Month 6), as well as with GPs in the clinic (–50.0% at Month 6) and non-face-to-face GP consultations (–62.0% at Month 6) (Supplementary Materials Table S8). Decreased costs were driven primarily by significant reductions in the mean number of HCP consultations at Month 3 (–55% [95% CI: –64%, –44%]; *p* < 0.001) and at Month 6 (–66% [95% CI: –74%, –55%]; *p* < 0.001) (Supplementary Materials Table S9). Notable reductions were observed in the number of consultations with stoma nurses in the clinic (–79.9% at Month 6) and non-face-to-face nurse consultations (–76.2% at Month 6), as well as with GPs in the clinic (–58.2% at Month 6) and non-face-to-face GP consultations (–68.6% at Month 6) (Supplementary Materials Table S9).

Costs associated with stoma-related hospitalisations decreased from baseline by 62.2% at Month 3 (−£1441 [95% CI: −£3486, £605]; *p* = 0.165) and by 81.3% at Month 6 (−£1885 [95% CI: −£3970, £201]; *p* = 0.076) but were not statistically significant (Table 1). Twenty percent of patients (*n* = 20/98) had ≥1 hospital admission at baseline, compared with only 12% at Month 3 (*n* = 11/95) and at Month 6 (*n* = 10/84).

### 3.2. Ostomy Solution Costs

**Figure 1:**
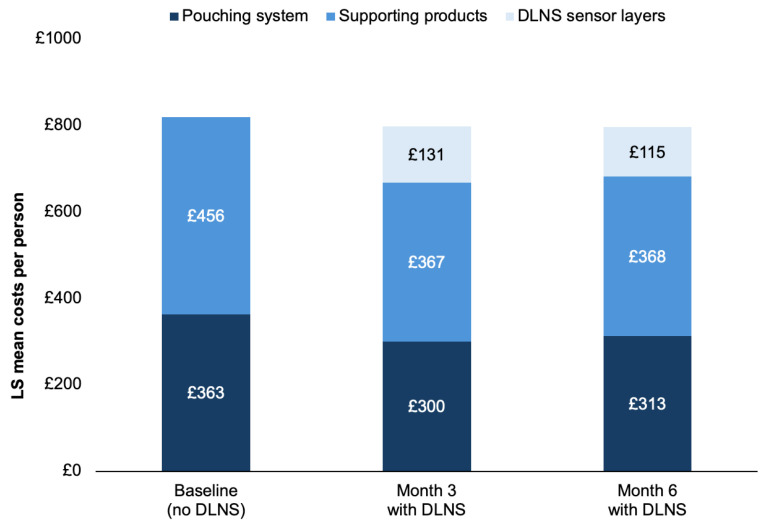
LS mean ostomy solution costs. DLNS, digital leakage notification system; LS, least squares.

The LS mean pouching system costs decreased significantly from baseline by 17.6% at Month 3 (−£64 [95% CI: −£98, −£30]; *p* < 0.001) and by 13.8% at Month 6 (−£50 [95% CI: −£85, −£16]; *p* = 0.004) (Figure 1; Table 2). Supporting product costs decreased significantly from baseline by 19.5% at Month 3 (−£89 [95% CI: −£126, −£52]; *p* < 0.001) and by 19.3% at Month 6 (−£88 [95% CI: −£131, −£45]; *p* < 0.001) (Table 2). Reductions were observed across multiple categories of supporting products, notably for adhesive tape, adhesive remover wipes, skin cleanser wipes, and paste (Supplementary Materials Table S10). The estimated mean DLNS sensor layer costs were £131 (SE, £11) at Month 3 and £115 (SE, £11) at Month 6, based on the mean reported use of 42 (SD, 34) and 37 (SD, 36) sensor layers per 3-month period, respectively. Total ostomy solution costs per person remained stable following DLNS initiation, with nominal, non-significant reductions from baseline of 1% at Month 3 (−£4 [95% CI: −£69, £61]; *p* = 0.897) and 0.2% at Month 6 (−£2 [95% CI: −£74, £70]; *p* = 0.955), as DLNS sensor layer costs were offset by reductions in other ostomy solution costs (Table 2).

**Figure 2:**
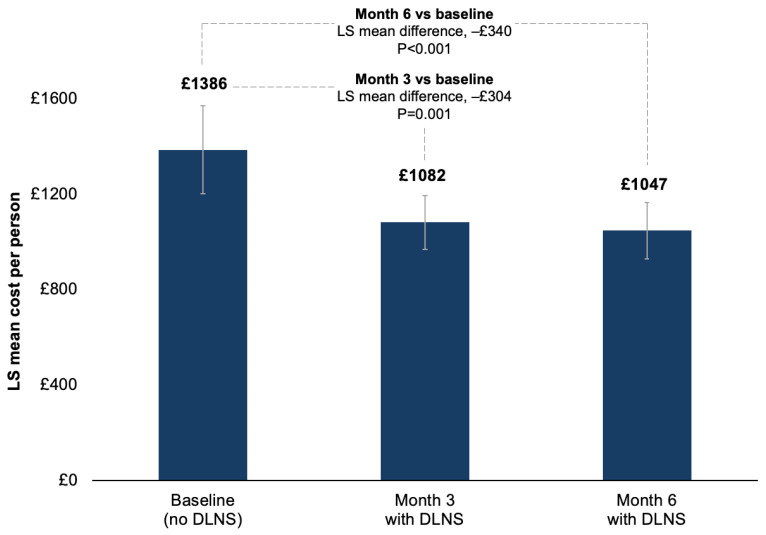
Total estimated LS mean costs, including stoma-related HCP consultations and the ostomy solution. HCP, healthcare provider; LS, least squares. Error bars represent 95% confidence intervals of the LS means.

The total estimated LS mean costs of stoma-related HCP consultations plus ostomy solution costs decreased significantly from baseline by 21.9% at Month 3 (−£304 [95% CI: −£456, −£153]; *p* = 0.001) and by 24.5% at Month 6 (−£340 [95% CI: −£504, −£175]; *p* < 0.001) (Figure 2).

### 3.3. Sensitivity Analyses

The sensitivity analyses assessing the impact of imputations applied to the pouching system and supporting product costs showed that the adjustments had a minimal influence on pouching system costs, with differences of only £4 (£367 vs. £363), £5 (£305 vs. £300), and £6 (£319 vs. £313) at baseline, Month 3 and Month 6, respectively. Supporting product costs were more affected, with reductions of £269 (£725 vs. £456), £177 (£544 vs. £367), and £210 (£578 vs. £368) across the three timepoints. These changes resulted in differences of £247 (£1030 vs. £784), £176 (£955 vs. £779), and £208 (£989 vs. £782) in total ostomy solution costs at baseline, Month 3, and Month 6, respectively. Overall, the sensitivity analysis indicated that the applied adjustments used in the primary analysis provided more conservative, and, therefore, more robust, estimates of costs and cost savings (Supplementary Materials Table S11).

### 3.4. Exploratory Subgroup Analysis

**Figure 3:**
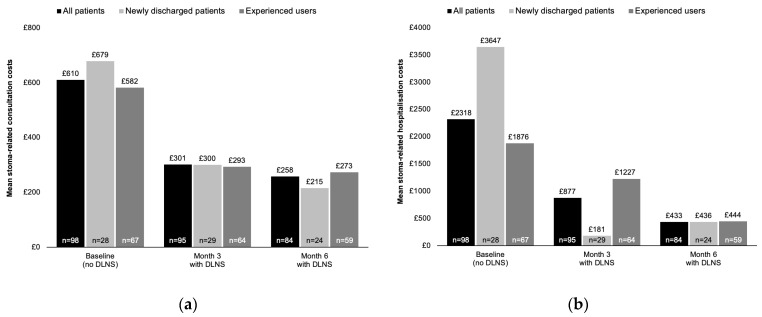
Exploratory subgroup analysis results. (**a**) Impact of time since stoma surgery on stoma-related HCP consultation costs; (**b**) impact of time since surgery on stoma-related hospitalisation costs. Figures should be placed in the main text near the first time they are cited. Baseline values are the estimated LS mean costs. Sample means were analysed for the Month 3 and Month 6 timepoint costs. Newly discharged patients were categorised as those whose stoma surgery occurred within 12 months before baseline; experienced users were those whose stoma surgery occurred >12 months before baseline.

In the exploratory analysis evaluating whether time since stoma surgery influenced the costs of stoma-related HCP consultations and hospitalisations, newly discharged patients (stoma surgery in the past 12 months) incurred slightly higher costs at baseline than experienced patients (stoma surgery >1 year ago; £679 vs. £582). Costs were roughly similar between groups at Month 3 and marginally higher among experienced users at Month 6 (£273 vs. £215) (Figure 3a). Stoma-related hospitalisation costs were notably higher among newly discharged patients compared with experienced users at baseline (£3647 vs. £1876), higher among experienced users at Month 3 (£181 vs. £1227), and appeared to be comparable between groups at Month 6 (£436 vs. £444, respectively) (Figure 3b).

## 4. Discussion

This costing analysis provides real-world evidence on the economic impact of using the Heylo™ DLNS for people living with a stoma in the UK based on the planned interim analysis of the first 100 participants with follow-up data at Month 6 of an ongoing longitudinal observational study [18]. The DLNS was associated with significant reductions in stoma-related HCP consultations and costs, with a trend toward reduced hospitalisation costs, and was cost-neutral in terms of total ostomy solution costs, including costs of the DLNS sensor layers after initiation of the DLNS. The largest and most consistent cost savings were those for stoma-related HCP consultations, which decreased from baseline by 51% (LS mean, −£309) at Month 3 and 59% (−£353) at Month 6, driven by fewer visits with both stoma care nurses and GPs. In this context, it should be noted that even modest reductions in demand for community and primary care providers may free up clinical capacity, aligning with national priorities to alleviate NHS workforce pressures, such as those identified in the NHS Long Term Workforce Plan [30] and the 10-year plan for England: Fit for the Future [31], which emphasise digital innovation and preventive, community-based care.

Total ostomy solution costs remained stable following initiation of the DLNS, with nominal, non-significant mean reductions from baseline of −£4 and −£2 per person at Months 3 and 6, where reductions in pouching system and supporting product costs offset the additional cost of DLNS sensor layers. The lower costs of supporting products observed at Month 6 likely reflect reduced worry about leakage and fewer unplanned pouch changes, consistent with reports of worry-related stoma product use [7], fewer leakage events, and improved leakage-related quality of life with DLNS use [7,14,18]. Although not statistically significant, the LS mean stoma-related hospitalisation costs decreased from baseline by 62% at Month 3 and 81% at Month 6; these estimates are expected to be more precise when the study is completed. Nonetheless, since hospitalisations are among the most expensive aspects of stoma care, even a numerical reduction may have a meaningful system-level impact. Taken together, these findings suggest that the DLNS may help to reduce economic and healthcare workforce capacity pressures while enabling more proactive patient management of leakages and related complications, while providing users with a significant improvement to their quality of life.

The exploratory analysis supported the primary findings and provided additional context, where time since surgery did not show substantial differences over time, though newly discharged patients had higher HCP consultation and hospitalisation costs at baseline, consistent with evidence that the first year after stoma formation carries the highest risk of complications [32]. Small subgroup sizes, however, limit interpretation, and further evaluation is warranted in the full cohort.

These findings complement a previously published cost-effectiveness model of the DLNS by Boisen et al. (2025) that projected similar overall cost patterns but did not incorporate stoma-related hospitalisations and included the cost of the DLNS starter kit [24]. The present analysis used real-world resource utilisation and estimated lower baseline ostomy solution costs than the model (£1030 unadjusted; £784 after ruleset adjustments vs. £1547 in the model). Following conservative adjustment of high supporting product use, the current analysis suggests that the DLNS is likely cost-saving from a healthcare system perspective, strengthening and extending the conclusions of the modelling study.

Since participants in the observational study largely remained with their selected pouching systems, product switching was unlikely to have a meaningful influence on cost trends [18]. The analysis also identified biases in self-reported use of supporting products; however, the adjustments to the data on supporting product usage lowered the estimated supporting product costs at both baseline and follow-up, ensuring product use and associated costs were not overestimated. Because these adjustments reduced costs at baseline more than costs at the follow-up timepoints, the resulting cost differences should be considered conservative estimates.

Strengths of this analysis include the use of data from the prospective observational study, detailed costing across multiple domains, and the use of established UK tariff sources, enhancing relevance for NHS decision-makers. Granular data on pouching systems, supporting products, consultations, and admissions allowed a comprehensive assessment of the economic impact of DLNS use. Limitations include reliance on self-reported data on product use and healthcare interactions, which may have inherent recall bias. Extrapolating shorter recall periods over a longer timeframe may amplify measurement error, although conservative assumptions were applied to minimise potential overestimation. DLNS usage frequency may also have been underestimated if temporary factors may have limited use during the recall period. Upon study completion and analysis of the full dataset, these biases may be substantially reduced. As a single-arm, pre/post observational study, the analysis is subject to potential Hawthorne effects and temporal confounding, and, therefore, observed changes should not be interpreted as causal effects; they reflect real-world patterns rather than controlled intervention effects. Hospitalisation costs were estimated using ICD-10 and HRG mapping because the specific reasons for each participant’s admission were not collected. Although ICD-10 and HRG tariffs are precise when applied to individual episodes, using population-level code distributions to approximate costs for unclassified admissions introduces some uncertainty. As this analysis was based on the first 100 participants enrolled, the study population may not be fully representative of the broader population of people living with a stoma. In particular, participants may be more likely to experience or be worried about leakage, reflecting the intended use of the DLNS in individuals with higher leakage burden. Tools such as the Leakage Impact Assessment tool, which assess leakage-related concerns, may help to identify patients who are most likely to benefit from the DLNS in clinical practice. Representativeness will be further evaluated upon completion of enrolment and follow-up of the full study cohort [33]. Finally, subgroup analyses were limited by small sample sizes, and confidence intervals remained wide, especially for hospitalisation costs. The costing analysis excluded the DLNS starter kit because it is not currently charged to the NHS, ensuring that findings reflect real-world practice.

## 5. Conclusions

In this costing analysis based on 6-month interim findings from an ongoing observational study, the Heylo™ DLNS was associated with significant cost savings from reduced stoma-related HCP consultations and pouching system and supporting product use. These savings resulted in a neutral overall ostomy solution cost when the DLNS was added to the stoma care regimen. Together with previously reported clinical benefits, these findings suggest that the Heylo™ DLNS meaningfully reduces healthcare utilisation while improving patient outcomes during the first 6 months of use. Forthcoming 12-month results will further clarify longer-term cost trajectories and the potential for broader efficiency gains within the NHS.

## Figures and Tables

**Table 1:** Costs associated with stoma-related HCP visits and hospitalisations.

Cost Components	Baseline (*n* = 100)	3 Months (*n* = 95)	6 Months (*n* = 84)
All HCP visits			
LS mean (SE)	£610 (£75)	£301 (£33)	£258 (£31)
Median (quartiles)	£382 (£142, £792)	£234 (£60, £442)	£159 (£0, £408)
*LS mean difference (95% CI)*	–	**−£309 (−£447, −£171); *p* < 0.001**	***−£353 (−£493, −£213); p < 0.001***
All hospitalisations			
LS mean (SE)	£2318 (£1034)	£877 (£4823)	£433 (£146)
Median (quartiles)	£0	£0	£0
*LS mean difference (95% CI)*	–	**−£1441 (−£3486, £605); *p* = 0.165**	***−£1885 (−£3970, £201); p = 0.076***
All HCP visits			
LS mean (SE)	£610 (£75)	£301 (£33)	£258 (£31)
Median (quartiles)	£382 (£142, £792)	£234 (£60, £442)	£159 (£0, £408)
*LS mean difference (95% CI)*	–	**−£309 (−£447, −£171); *p* < 0.001**	***−£353 (−£493, −£213); p < 0.001***
All hospitalisations			
LS mean (SE)	£2318 (£1034)	£877 (£4823)	£433 (£146)
Median (quartiles)	£0	£0	£0
*LS mean difference (95% CI)*	–	**−£1441 (−£3486, £605); *p* = 0.165**	***−£1885 (−£3970, £201); p = 0.076***
All HCP visits			
LS mean (SE)	£610 (£75)	£301 (£33)	£258 (£31)
Median (quartiles)	£382 (£142, £792)	£234 (£60, £442)	£159 (£0, £408)
*LS mean difference (95% CI)*	–	**−£309 (−£447, −£171); *p* < 0.001**	***−£353 (−£493, −£213); p < 0.001***

CI, confidence interval; DLNS, digital leakage notification system; HCP, healthcare provider; LS, least squares; SE, standard error.

**Table 2:** Ostomy solution costs.

Cost Components	Baseline (n = 100)	3 Months (n = 95)	6 Months (n = 84)
Pouching system costs			
LS mean (SE)	£363 (£20)	£300 (£18)	£313 (£20)
Median (quartiles)	£325 (£228, £460)	£230 (£188, £382)	£260 (£190, £393)
*** LS mean difference (95% CI)*** ***compared to baseline***	–	***−£64 (−£98, −£30); p < 0.001***	***−£50 (−£85, −£16); p = 0.004***
Supporting product costs			
LS mean (SE)	£456 (£29)	£367 (£23)	£368 (£23)
Median (quartiles)	£394 (£234, £570)	£328 (£199, £450)	£320 (£229, £463)
*** LS mean difference (95% CI)*** ***compared to baseline***	–	***−£89 (−£126, −£52); p < 0.001***	***−£88 (−£131, −£45); p < 0.001***
DLNS sensor layer costs	–		
LS mean (SE)		£131 (£11)	£115 (£11)
Median (quartiles)		£121 (£53, £179)	£94 (£24, £181)
Total ostomy solution costs			
LS mean (SE)	£784 (£39)	£779 (£41)	£782 (£43)
Median (quartiles)	£799 (£521, £1033)	£682 (£497, £1006)	£715 (£519, £1023)
*** LS mean difference (95% CI)*** ***compared to baseline***	–	***−£4 (−£60, £61); p = 0.897***	***−£2 (−£74, £70); p = 0.955***

CI, confidence interval; DLNS, digital leakage notification system; HCP, healthcare provider; LS, least squares; SE, standard error.

## Data Availability

The original contributions presented in this study are included in the article/Supplementary Materials. Further inquiries can be directed to the corresponding author(s).

## Supplementary Materials

The following supporting information can be downloaded at: https://www.mdpi.com/article/10.3390/jmahp14020029/s1, Table S1: One-piece pouching system unit costs [21,24]; Table S2: Two-piece pouching system unit costs [21,24]; Table S3: Supporting product costs weighted by market share [21,24]; Table S4: Weighted cost of belts based on market share [26]; Table S5: Healthcare provider consultation costs [22,23]; Table S6: Top 20 reasons for hospitalisations and HRG code match [27]; Table S7: Activities and costs associated with relevant HRGs; Table S8: Estimated 3-month individual and total costs of stoma-related consultations (£); Table S9: Estimated 3-month stoma-related consultations; Table S10: Estimated 3-month costs of supporting products (£); Table S11: Sensitivity analysis adjusted vs. unadjusted ostomy product and solution costs (£). Table S12: The official Bank of England exchange rates from GBP to EUR and USD for the date that the costings analysis was completed, 15th September 2025.

## Abbreviations

The following abbreviations are used in this manuscript:

CI	Confidence interval
DLNS	Digital leakage notification system
ECDS	Emergency Care Dataset
GP	General practitioner
HCP	Healthcare professional
HES	Hospital Episode Statistics
HRG	Health Resource Group
LS	Least squares
NHS	National Health Service
PSSRU	Personal Social Services Research Unit
QoL	Quality of life
SD	Standard deviation
SE	Standard error
