# Supplementary material for: Impact of a Digital Leakage Notification System on Healthcare Resource Utilisation and Costs for People with Stomas in the United Kingdom: Analysis of Interim Results from a Prospective Longitudinal Study
Source: J Mark Access Health Policy. 2026 May 6;14(2):29. doi: 10.3390/jmahp14020029 (PMC13214903; doi:10.3390/jmahp14020029)
Supplement: Supplementary file 1 [file jmahp-14-00029-s001.zip › jmahp-4190183-supplementary.pdf]

Impact of a digital leakage notification system on healthcare resource utilisation and costs for people with stomas in the United Kingdom: Analysis of interim results from a prospective longitudinal study

Alexandra Baxter[1], Esben Bo Boisen[1], Majken Linnemann Jensen[1], Aamir Shaikh[2]

[1]Coloplast A/S, Humlebæk, Denmark; [2]Coloplast Ltd, Peterborough, United Kingdom

Supplementary Materials

Table S1. One-piece pouching system unit costs[1,2]

	Drainable systems		Closed systems	
**System (Manufacturer)**	**Unit cost**	**Patients (%)**	**Unit cost**	**Patients (%)**
**Flat baseplates**				
SenSura® Mio (Coloplast)	£3.61	53.1%	£3.22	53.1%
Nova™ 1 (Dansac)	£3.29	26.7%	£2.88	26.7%
Moderma Flex™ Cera Plus™ (Hollister)	£3.80	3.9%	£3.16	3.9%
Confidence® (Salts)	£3.58	14.4%	£3.19	14.4%
Esteem® Plus (Convatec)	£3.51	1.9%	£3.12	1.9%
SoC basket of comparators	£3.53	100.0%	£3.12	100.0%
**Convex baseplates**				
SenSura® Mio (Coloplast)	£4.97	44.7%	£3.60	44.7%
NovaLife™ TRE (Dansac)	£5.16	17.4%	£3.47	17.4%
Moderma Flex™ Cera Plus™ (Hollister)	£5.15	11.8%	£3.56	11.8%
Confidence® Natural (Salts)	£4.87	23.6%	£3.28	23.6%
Esteem® Plus Soft (Convatec)	£4.56	2.4%	£3.28	2.4%
SoC basket of comparators	£4.99	100.0%	£3.49	100.0%
**Concave baseplates**				
SenSura® Mio (Coloplast)	£5.04	100.0%	£3.65	100.0%

Assumptions were identical for patients with ileostomy or colostomy.

Table S2. Two-piece pouching system unit costs[1,2]

System (Manufacturer)	Unit cost	Patients (%)
**Flat baseplates**		
SenSura® Mio (Coloplast)	£4.27	14.3%
Nova™ 2 (Dansac)	£3.54	25.8%
Conform 2™ (Hollister)	£3.43	11.4%
Harmony® Duo (Salts)	£3.97	3.7%
Combihesive Natura® (Convatec)	£3.69	44.9%
SoC basket of comparators	£3.72	100.0%
**Convex baseplates**		
SenSura® Mio Flex (Coloplast)	£4.38	20.5%
Nova™ 2 (Dansac)	£3.61	42.6%
Conform 2™ CeraPlus™ (Hollister)	£4.99	17.6%
Harmony® Duo (Salts)	£4.25	2.4%
Combihesive Natura® (Convatec)	£3.77	16.9%
SoC basket of comparators	£4.05	100.0%
**Concave baseplates**		
SenSura® Mio (Coloplast)	£4.42	100.0%

Assumptions were identical for patients with ileostomy or colostomy.

Table S3. Supporting product costs weighted by market share[1,2]

Supporting product	Product name	Cost	Market share split	Weighted average
Belt	Brava belt for SenSura Mio standard (Coloplast)	£7.52	94%	£7.52
	Brava belt for SenSura Mio XX large (Coloplast)	£7.44	6%	
	Adapt ostomy belt 58cm-109cm (Hollister)	£8.86	74%	£8.86
	Adapt ostomy belt 86cm-165cm (Hollister)	£8.86	26%	
	Ostomy belt long 150cm white (Dansac)	£8.63	11%	£8.63
	Ostomy belt long beige 100cm (Dansac)	£8.63	76%	
	Ostomy belt long beige 150cm (Dansac)	£8.63	13%	
	Adjustable ostomy belt (Salts)	£8.55	97%	£8.55
	Adjustable ostomy belt 122cm beige (Salts)	£8.47	3%	
	Adjustable ostomy belt (Convatec)	£3.93	100%	£3.93
Paste	Coloplast Brava Strip Paste	£0.87	100%	£0.87
Tape	Brava elastic tape	£0.72	81%	£0.73
	Brava elastic tape belt	£0.74	4%	
	Brava elastic tape straight	£0.74	1%	
	Brava elastic tape XL	£0.83	14%	
Skin protection	Brava Coloplast protective sheet 10cm x 10cm	£2.70	3%	£0.60
	Brava Coloplast protective sheet 15cm x 15cm	£6.46	1%	
	Brava Coloplast protective sheet 20cm x 20cm	£11.75	0%	
	Brava skin barrier cream (per ml)	£0.09	16%	
	Brava skin barrier spray (per ml)	£0.26	40%	
	Brava skin barrier wipes	£0.84	40%	
Adhesive remover	Brava adhesive remover spray	£10.18	95%	£9.80
	Brava adhesive remover spray XL	£10.49	1%	
	Brava adhesive remover wipes	£0.56	5%	
Ring/seal	Brava mouldable ring 48mm x 2mm	£2.12	10%	£2.19
	Brava mouldable ring 48mm x 4.2mm	£2.22	4%	
	Brava protective seal 18-48mm 2.5mm	£2.10	23%	
	Brava protective seal 18-48mm 4.2mm	£2.13	2%	
	Brava protective seal 18-57mm 2.5mm	£2.24	6%	
	Brava protective seal 18-57mm 4.2mm	£2.30	0%	
	Brava protective seal 18-64mm 2.5mm	£2.28	4%	
	Brava protective seal 18-64mm 4.2mm	£2.36	1%	
	Brava protective seal 18-76mm 2.5mm	£2.48	2%	
	Brava protective seal 27-57mm 2.5mm	£2.17	25%	
	Brava protective seal 27-57mm 4.2mm	£2.24	3%	
	Brava protective seal 27-76mm 2.5mm	£2.48	4%	
	Brava protective seal 36-64mm 2.5mm	£2.22	13%	
	Brava protective seal 36-64mm 4.2mm	£2.29	2%	
Skin cleaner	Brava Skin Cleanser Wipe	£0.39	100%	£0.39
Ostomy powder	Coloplast Brava Ostomy Powder	£2.64	100%	£2.64
Deodorant/lubrication	Coloplast Brava Lubricating Deodorant Sachet	£0.25	0%	£9.20
	Coloplast Brava Lubricating Deodorant	£9.20	100%	
Heylo™	Heylo™ Sensor Layer	£3.09	100%	£3.09

Table S4. Weighted cost of belts based on market share[3]

Manufacturer	Market share	Basket cost
Coloplast	48%	£7.80
Hollister	7%	
Dansac	24%	
Salts	15%	
Convatec	6%	

Table S5. Healthcare provider consultation costs[4,5]

Type of consultation	Cost	Source
Stoma Care Nurse in clinic	£60	N24AF Specialist Nursing, Stoma Care Service, adult, face-to-face; Weighted average of service codes 07, 12, 15, 22, 29, 52; per appointment[4]
Stoma Care Nurse at home	£57	N24AN Specialist Nursing, Stoma Care Service, adult, non–face-to-face; Weighted average of service codes 07, 12, 15, 22, 29, 52; per appointment[4]
Home Care Nurse (district nurse)	£57	N02AF District Nurse Adult, face-to-face; service code 12; per appointment[4]
Nurse (district nurse), non-face-to-face (non-Coloplast/non-charter)	£38	N02AN District Nurse Adult, non–face-to-face; 12 District Nursing; per appointment[4]
General Practitioner, face-to-face	£243	General Practitioner, unit cost per hour of patient context, excluding direct care staff and including qualification costs[5]
General Practitioner, non-face-to-face	£243	General Practitioner, unit cost per hour of patient context, excluding direct care staff and including qualification costs[5]
Dermatologist, face-to-face	£177	Consultant-led outpatient appointment service code: 330; WF01B; per appointment[4]
Gastroenterologist, face-to-face	£238	Consultant-led outpatient appointment service code: 301; WF01B; per appointment[4]
Stoma Surgeon, face-to-face	£211	Consultant-led outpatient appointment service code: 100; WF01B; per appointment[4]

Table S6. Top 20 reasons for hospitalisations and HRG code match[6]

ICD-10 Code	Code Name	Activities (2023-2024)	Activities (2024-2025)	Activities (Total)	Matched HRG Code	Code Description
K435	Parastomal hernia without obstruction or gangrene	2415	2375	4790	FD10	Non-Malignant Gastrointestinal Tract Disorders
K433	Parastomal hernia with obstruction, without gangrene	1890	1825	3715	FD10	Non-Malignant Gastrointestinal Tract Disorders
C20X	Malignant neoplasm of rectum	1340	1160	2500	FD11	Malignant Gastrointestinal Tract Disorders
K914	Colostomy and enterostomy malfunction	895	885	1780	FD10	Non-Malignant Gastrointestinal Tract Disorders
K632	Fistula of intestine	730	765	1495	FD10	Non-Malignant Gastrointestinal Tract Disorders
K566	Other and unspecified intestinal obstruction	715	680	1395	FD10	Non-Malignant Gastrointestinal Tract Disorders
K432	Incisional hernia without obstruction or gangrene	765	640	1405	FD10	Non-Malignant Gastrointestinal Tract Disorders
Z432	Attention to ileostomy	615	605	1220	FD10	Non-Malignant Gastrointestinal Tract Disorders
A419	Sepsis, unspecified	540	605	1145	WJ06	Sepsis with Single, Multiple & No Interventions
K565	Intestinal adhesions [bands] with obstruction	505	560	1065	FD10	Non-Malignant Gastrointestinal Tract Disorders
L022	Cutaneous abscess, furuncle and carbuncle of trunk	600	525	1125	JD07	Skin Disorders with Interventions, with CC Score 12+
C187	Malignant neoplasm: Sigmoid colon	895	480	1375	FD11	Malignant Gastrointestinal Tract Disorders
N390	Urinary tract infection, site not specified	465	460	925	LA04	Kidney or Urinary Tract Infections
T814	Infection following a procedure, not elsewhere classified	400	415	815	WH07	Infections or Other Complications of Procedures
N179	Acute renal failure, unspecified	450	405	855	LA07	Acute Kidney Injury with Interventions
J181	Lobar pneumonia, unspecified	455	400	855	DZ11	Lobar, Atypical or Viral Pneumonia,
C780	Secondary malignant neoplasm of lung	*Excluded due to non-relevance to stoma*				
K509	Crohn’s disease, unspecified	435	365	800	FD02	Inflammatory Bowel Disease with Multiple Interventions
E834	Disorders of magnesium metabolism	440	320	760	KC05	Fluid or Electrolyte Disorders, with Interventions

Table S7. Activities and costs associated with relevant HRGs

HRG Code	Total Activity	Total Cost	Average
FD10	414,975	£872,227,287	£2,101.88
FD11	49,561	£195,476,498	£3,944.16
WJ06	208,128	£719,478,363	£3,456.90
JD07	250,301	£547,683,292	£2,188.10
LA04	123,759	£486,888,874	£3,934.17
WH07	65,993	£157,340,420	£2,384.20
LA07	101,696	£279,261,427	£2,746.04
DZ11	522,577	£1,390,332,444	£2,660.53
FD02	59,704	£141,773,560	£2,374.61
KC05	136,402	£270,670,123	£1,984.36
**Total**	**1,933,096**	**£5,061,132,288**	**£2,618.15**

HRG, Healthcare Resource Group.

Table S8. Estimated 3-month individual and total costs of stoma-related consultations (£)

Category	Statistic	Baseline (n=98)	Month 3 (n=95)	Month 6 (n=84)
**Total stoma-related consultation cost**	LS Mean (SE)	610 (75)	301 (33)	258 (31)
	95% CI (LS mean)	[461; 759]	[236; 367]	[197; 319]
	Median	382	234	159
	Quartiles (Q1; Q3)	[142; 792]	[60; 442]	[0; 408]
	Difference vs baseline, LS Mean (SE)	–	−309 (70)	−353 (71)
	95% CI	–	[−447; −171]	[−493; −213]
	P-value	–	<0.001	<0.001
**Stoma Care Nurse in clinic**	Mean (SD)	128 (149)	61 (73)	30 (54)
	Median	90	60	0
	Quartiles (Q1; Q3)	[60; 180]	[0; 120]	[0; 60]
**Stoma Care Nurse at home**	Mean (SD)	28 (71)	13 (46)	4 (19)
	Median	0	0	0
	Quartiles (Q1; Q3)	[0; 0]	[0; 0]	[0; 0]
**Home Care Nurse (district nurse)**	Mean (SD)	6 (37)	0 (0)	2 (14)
	Median	0	0	0
	Quartiles (Q1; Q3)	[0; 0]	[0; 0]	[0; 0]
**Nurse (district nurse), non-face-to-face**	Mean (SD)	67 (114)	21 (41)	21 (47)
	Median	38	0	0
	Quartiles (Q1; Q3)	[0; 76]	[0; 38]	[0; 38]
**General Practitioner, face-to-face**	Mean (SD)	92 (251)	47 (104)	46 (106)
	Median	0	0	0
	Quartiles (Q1; Q3)	[0; 61]	[0; 61]	[0; 61]
**General Practitioner, non-face-to-face**	Mean (SD)	50 (110)	27 (83)	19 (53)
	Median	0	0	0
	Quartiles (Q1; Q3)	[0; 61]	[0; 0]	[0; 0]
**Dermatologist, face-to-face**	Mean (SD)	13 (81)	9 (47)	6 (33)
	Median	0	0	0
	Quartiles (Q1; Q3)	[0; 0]	[0; 0]	[0; 0]
**Gastroenterologist, face-to-face**	Mean (SD)	92 (210)	63 (131)	65 (145)
	Median	0	0	0
	Quartiles (Q1; Q3)	[0; 0]	[0; 0]	[0; 0]
**Stoma Surgeon, face-to-face**	Mean (SD)	140 (307)	62 (115)	60 (111)
	Median	0	0	0
	Quartiles (Q1; Q3)	[0; 211]	[0; 106]	[0; 53]

CI, confidence interval; LS, least squares; Q, quartile; SD, standard deviation; SE, standard error.

Table S9. Estimated 3-month stoma-related consultations

Category	Statistic	Baseline (n=98)	Month 3 (n=95)	Month 6 (n=84)
**Total stoma-related consultation frequency**	Total consultations	717	313	210
	LS Mean	7.3	3.3	2.5
	95% CI	[5.9; 9.0]	[2.7; 3.9]	[2.0; 3.1]
	Reduction vs baseline	–	55%	66%
	95% CI (reduction)	–	[44%; 64%]	[55%; 74%]
	P-value	–	<0.001	<0.001
**Stoma Care Nurse, clinic**	Total consultations	209	97	42
	LS Mean	2.1	1	0.5
	95% CI	[1.7; 2.7]	[0.8; 1.3]	[0.3; 0.7]
**Stoma Care Nurse, home**	Total consultations	48	21	6
	LS Mean	0.5	0.2	0.1
	95% CI	[0.3; 0.8]	[0.1; 0.5]	[0.0; 0.2]
**Home Care Nurse (district nurse)**	Total consultations	10	0	3
	LS Mean	Not estimated	Not estimated	Not estimated
**Nurse, non-face-to-face**	Total consultations	172	52	41
	LS Mean	1.8	0.5	0.6
	95% CI	[1.2; 2.5]	[0.4; 0.8]	[0.3; 0.9]
**General Practitioner, face-to-face**	Total consultations	98	55	41
	LS Mean	1	0.6	0.5
	95% CI	[0.6; 1.6]	[0.4; 0.8]	[0.3; 0.7]
**General Practitioner, non-face-to-face**	Total consultations	70	30	22
	LS Mean	0.7	0.3	0.3
	95% CI	[0.5; 1.1]	[0.2; 0.6]	[0.2; 0.4]
**Dermatologist, face-to-face**	Total consultations	7	5	3
	LS Mean	Not estimated	Not estimated	Not estimated
**Gastroenterologist, face-to-face**	Total consultations	38	25	23
	LS Mean	0.4	0.3	0.3
	95% CI	[0.3; 0.6]	[0.2; 0.4]	[0.2; 0.5]
**Stoma Surgeon, face-to-face**	Total consultations	65	28	24
	LS Mean	0.7	0.3	0.3
	95% CI	[0.4; 1.0]	[0.2; 0.4]	[0.2; 0.4]

CI, confidence interval; LS, least squares.

Table S10. Estimated 3-month costs of supporting products (£)

Product	Statistic	Baseline (n=98)	Month 3 (n=95)	Month 6 (n=84)
**Total Supporting Products**	LS Mean (SE)<br>[95% CI of LS mean]<br>Median<br>Quartiles [Q1: Q3]	456 (29)<br>[399; 513]<br>394<br>[234; 570]	367 (23)<br>[322; 412]<br>328<br>[199; 450]	368 (23)<br>[323; 414]<br>320<br>[229; 463]
	Diff. of LS means (SE)<br>[95% CI]<br>P-value	N/A	-89 (18.59)<br>[-126; -52]<br>P<0.001	-88 (22)<br>[-131; -45]<br>P<0.001
Rings/Seals	Mean (SD)<br>Median<br>Quartiles [Q1: Q3]	114 (121)<br>86<br>[0: 200]	96 (107)<br>71<br>[0: 143]	96 (95)<br>86<br>[0: 143]
Paste (tubes)	Mean (SD)<br>Median<br>Quartiles [Q1: Q3]	13 (32)<br>0<br>[0: 0]	10 (£28)<br>0<br>[0: 0]	10.13 (28)<br>0<br>[0: 0]
Paste (strips)	Mean (SD)<br>Median<br>Quartiles [Q1: Q3]	5 (22)<br>0<br>[0: 0]	3 (17)<br>0<br>[0: 0]	2 (13)<br>0<br>[0: 0]
Adhesive tape (edge tape)	Mean (SD)<br>Median<br>Quartiles [Q1: Q3]	41 (51)<br>19<br>[0: 67]	26 (42)<br>0<br>[0: 38]	29 (43)<br>0<br>[0: 48]
Adhesive remover, spray	Mean (SD)<br>Median<br>Quartiles [Q1: Q3]	107 (36)<br>133<br>[66: 133]	96 (37)<br>66<br>[66: 133]	100 (36)<br>133<br>[66: 133]
Adhesive remover, wipe	Mean (SD)<br>Median<br>Quartiles [Q1: Q3]	26 (44)<br>0<br>[0: 47]	17 (30)<br>0<br>[0: 30]	15 (27)<br>0<br>[0: 33]
Skin cleanser, wipes	Mean (SD)<br>Median<br>Quartiles [Q1: Q3]	28 (31)<br>18<br>[0: 51]	23 (26)<br>18<br>[0: 36]	20 (25)<br>9<br>[0: 29]
Skin barrier, spray	Mean (SD)<br>Median<br>Quartiles [Q1: Q3]	30 (59)<br>0<br>[0: 0]	26 (52)<br>0<br>[0: 0]	30 (58)<br>0<br>[0: 0]
Skin barrier, wipes	Mean (SD)<br>Median<br>Quartiles [Q1: Q3]	24 (42)<br>0<br>[0: 38]	16 (30)<br>0<br>[0: 27]	23 (41)<br>0<br>[0: 38]
Skin barrier cream, bottles	Mean (SD)<br>Median<br>Quartiles [Q1: Q3]	4 (16)<br>0<br>[0: 0]	4 (15)<br>0<br>[0: 0]	3 (12)<br>0<br>[0: 0]
Skin barrier sheet	Mean (SD)<br>Median<br>Quartiles [Q1: Q3]	20 (87)<br>0<br>[0: 0]	17 (81)<br>0<br>[0: 0]	14 (68)<br>0<br>[0: 0]
Belt	Mean (SD)<br>Median<br>Quartiles [Q1: Q3]	8 (8)<br>0<br>[0: 16]	7 (8)<br>0<br>[0: 16]	7 (8)<br>0<br>[0: 16]
Ostomy powder, bottles	Mean (SD)<br>Median<br>Quartiles [Q1: Q3]	10 (12)<br>0<br>[0: 17]	9 (12)<br>0<br>[0: 17]	9 (11)<br>0<br>[0: 17]
Deodorant, sachets	Mean (SD)<br>Median<br>Quartiles [Q1: Q3]	3 (10)<br>0<br>[0: 0]	4 (11)<br>0<br>[0: 0]	4 (12)<br>0<br>[0: 0]
Deodorant, bottles	Mean (SD)<br>Median<br>Quartiles [Q1: Q3]	18 (35)<br>0<br>[0: 0]	12 (30)<br>0<br>[0: 0]	14 (30)<br>0<br>[0: 0]

CI, confidence interval; LS, least squares; Q, quartile; SD, standard deviation; SE, standard error.

Table S11. Sensitivity analysis adjusted vs unadjusted ostomy product and solution costs (£)

Cost Category		Baseline		Month 3		Month 6	
		Unadjusted	Adjusted	Unadjusted	Adjusted	Unadjusted	Adjusted
Pouching system	N	89	93	87	90	77	79
	LS Mean<br>95% CI	367<br>[328; 407]	363<br>[324; 403]	305<br>[269; 342]	300<br>[264; 336]	319<br>[278; 360]	313<br>[273; 353]
	Diff. of LS Means<br>P-value	N/A		-62<br>P<0.001	-64<br>P<0.001	-49<br>P=0.006	-50<br>P=0.004
All supporting products	N	99	99	95	95	85	85
	LS Mean<br>95% CI	725<br>[580; 871]	456<br>[399; 513]	544<br>[463; 625]	367<br>[322; 412]	578<br>[467; 689]	368<br>[323; 414]
	Diff. of LS Means<br>P-value	N/A		-181<br>P=0.006	-89<br>P<0.001	-147<br>P=0.020	-83<br>P<0.001
Total ostomy solution	N	100	100	95	95	84	84
	LS Mean<br>95% CI	1030<br>[873; 1188]	784<br>[706; 861]	955<br>[845; 1065]	779<br>[698; 861]	989<br>[845; 1133]	782<br>[696; 867]
	Diff. of LS Means<br>P-value	N/A		-75<br>P=0.249	-4<br>P=0.897	-41<br>P=0.550	-2<br>P=0.955

CI, confidence intervall; LS, least squares.

Table S12 : The official Bank of England exchange rates from GBP to EUR and USD for the date that the costings analysis was completed, 15th September 2025.

Article section	Cost datapoint	GBP amount	Corresponding amount in EUR	Corresponding amount in USD
Abstract	Decrease from baseline to month 3 in total LS mean costs for stoma-related consultations plus ostomy solution use	-£304.00	-£351.39	-£413.14
Abstract	Lower limit of 95% confidence interval	-£456.00	-£527.09	-£619.70

Abstract	for the above Upper limit of 95% confidence interval for the above	-£153.00	-£176.85	-£207.93
Abstract	Decrease from baseline to month 6 in total LS mean costs for stoma-related consultations plus ostomy solution use	-£340.00	-£393.01	-£462.06
Abstract	Lower limit of 95% confidence interval for the above	-£504.00	-£582.57	-£684.94
Abstract	Upper limit of 95% confidence interval for the above	-£175.00	-£202.28	-£237.83
Abstract	Decrease from baseline to month 3 in total LS mean costs for stoma-related HCP consultations	-£309.00	-£357.17	-£419.93
Abstract	Lower limit of 95%	-£447.00	-£516.69	-£607.47

	confidence interval for the above			
Abstract	Upper limit of 95% confidence interval for the above	-£171.00	-£197.66	-£232.39
Abstract	Decrease from baseline to month 6 in total LS mean costs for stoma-related HCP consultations	-£353.00	-£408.03	-£479.73
Abstract	Lower limit of 95% confidence interval for the above	-£493.00	-£569.86	-£669.99
Abstract	Upper limit of 95% confidence interval for the above	-£213.00	-£246.21	-£289.47
Abstract	Mean ostomy solution costs per patient at 3 months	£784.00	£906.23	£1,065.46
Abstract	Mean ostomy solution costs per patient at 6 months	£782.00	£903.91	£1,062.74
Materials and	Total average cost of a	£2,618.15	£3,026.32	£3,558.07

methods, 173	stoma-related hospital admission			
Results, 200	Decrease from baseline to month 3 in total LS mean costs for stoma-related HCP consultations	-£309.00	-£357.17	-£419.93
Results, 200	Lower limit of 95% confidence interval for the above	-£447.00	-£516.69	-£607.47
Results, 200	Upper limit of 95% confidence interval for the above	-£171.00	-£197.66	-£232.39
Results, 201	Decrease from baseline to month 6 in total LS mean costs for stoma-related HCP consultations	-£353.00	-£408.03	-£479.73
Results, 201	Lower limit of 95% confidence interval for the above	-£493.00	-£569.86	-£669.99
Results, 201	Upper limit of	-£213.00	-£246.21	-£289.47

	95% confidence interval for the above			
Results, 213	Decrease from baseline to month 3 in total LS mean costs for stoma-related hospitalisations	-£1,441.00	-£1,665.65	-£1,958.32
Results, 213	Lower limit of 95% confidence interval for the above	-£3,486.00	-£4,029.47	-£4,737.47
Results, 213	Upper limit of 95% confidence interval for the above	£605.00	£699.32	£822.20
Results, 214	Decrease from baseline to month 6 in total LS mean costs for stoma-related hospitalisations	-£1,885.00	-£2,178.87	-£2,561.72
Results, 214	Lower limit of 95% confidence interval for the above	-£3,970.00	-£4,588.92	-£5,395.23
Results, 214	Upper limit of 95% confidenc	£201.00	£232.34	£273.16

	e interval for the above			
Table 1	LS mean of all HCP visits (baseline)	£610.00	£705.10	£828.99
Table 1	SE of the above	£75.00	£86.69	£101.93
Table 1	LS mean of all HCP visits (at 3 months)	£301.00	£347.93	£409.06
Table 1	SE of the above	£33.00	£38.14	£44.85
Table 1	LS mean of all HCP visits (at 6 months)	£258.00	£298.22	£350.62
Table 1	SE of the above	£31.00	£35.83	£42.13
Table 1	Median of all HCP visits (baseline)	£382.00	£441.55	£519.14
Table 1	Lower quartile limit of the above	£142.00	£164.14	£192.98
Table 1	Upper quartile limit of the above	£792.00	£915.47	£1,076.33
Table 1	Median of all HCP visits (at 3 months)	£234.00	£270.48	£318.01
Table 1	Lower quartile limit of the above	£60.00	£69.35	£81.54
Table 1	Upper quartile limit of the above	£442.00	£510.91	£600.68
Table 1	Median of all HCP visits (at 6 months)	£159.00	£183.79	£216.08
Table 1	Lower quartile	£0.00	£0.00	£0.00

Table 1	limit of the above Upper quartile limit of the above	£408.00	£471.61	£554.47
Table 1	LS mean difference for all HCP visits (at 3 months)	-£309.00	-£357.17	-£419.93
Table 1	Lower limit of 95% confidence interval for the above	-£447.00	-£516.69	-£607.47
Table 1	Upper limit of 95% confidence interval for the above	-£171.00	-£197.66	-£232.39
Table 1	LS mean difference for all HCP visits (at 6 months)	-£353.00	-£408.03	-£479.73
Table 1	Lower limit of 95% confidence interval for the above	-£493.00	-£569.86	-£669.99
Table 1	Upper limit of 95% confidence interval for the above	-£213.00	-£246.21	-£289.47
Table 1	LS mean of all stoma-related hospitalis	£2,318.00	£2,679.38	£3,150.16

	ations (baseline)			
Table 1	SE of the above	£1,034.00	£1,195.20	£1,405.21
Table 1	LS mean of all stoma-related hospitalisations (at 3 months)	£877.00	£1,013.72	£1,191.84
Table 1	SE of the above	£4,823.00	£5,574.91	£6,554.46
Table 1	LS mean of all stoma-related hospitalisations (at 6 months)	£433.00	£500.50	£588.45
Table 1	SE of the above	£146.00	£168.76	£198.41
Table 1	Median of all stoma-related hospitalisations (baseline)	£0.00	£0.00	£0.00
Table 1	Lower quartile limit of the above	£0.00	£0.00	£0.00
Table 1	Upper quartile limit of the above	£0.00	£0.00	£0.00
Table 1	Median of all stoma-related hospitalisations (at 3 months)	£0.00	£0.00	£0.00
Table 1	Lower quartile limit of the above	£0.00	£0.00	£0.00
Table 1	Upper quartile limit of the above	£0.00	£0.00	£0.00
Table 1	Median of all stoma-	£0.00	£0.00	£0.00

	related hospitalisations (at 6 months)			
Table 1	Lower quartile limit of the above	£0.00	£0.00	£0.00
Table 1	Upper quartile limit of the above	£0.00	£0.00	£0.00
Table 1	LS mean difference for all stoma-related hospitalisations (at 3 months)	-£1,441.00	-£1,665.65	-£1,958.32
Table 1	Lower limit of 95% confidence interval for the above	-£3,486.00	-£4,029.47	-£4,737.47
Table 1	Upper limit of 95% confidence interval for the above	£605.00	£699.32	£822.20
Table 1	LS mean difference for all stoma-related hospitalisations (at 6 months)	-£1,885.00	-£2,178.87	-£2,561.72
Table 1	Lower limit of 95% confidence interval for the above	-£3,970.00	-£4,588.92	-£5,395.23
Table 1	Upper limit of 95%	£201.00	£232.34	£273.16

	confidence interval for the above			
Results section, 222	Reduction in LS mean of pouching system costs (at 3 months)	-£64.00	-£73.98	-£86.98
Results section, 222	Lower limit of 95% confidence interval for the above	-£98.00	-£113.28	-£133.18
Results section, 222	Upper limit of 95% confidence interval for the above	-£30.00	-£34.68	-£40.77
Results section, 222	Reduction in LS mean of pouching system costs (at 3 months)	-£50.00	-£57.80	-£67.95
Results section, 223	Lower limit of 95% confidence interval for the above	-£85.00	-£98.25	-£115.52
Results section, 223	Upper limit of 95% confidence interval for the above	-£16.00	-£18.49	-£21.74
Results section, 224	Reduction in LS mean of supporting product	-£89.00	-£102.88	-£120.95

	costs (at 3 months)			
Results section, 224	Lower limit of 95% confidence interval for the above	-£126.00	-£145.64	-£171.23
Results section, 224	Upper limit of 95% confidence interval for the above	-£52.00	-£60.11	-£70.67
Results section, 225	Reduction in LS mean of supporting product costs (at 6 months)	-£88.00	-£101.72	-£119.59
Results section, 225	Lower limit of 95% confidence interval for the above	-£131.00	-£151.42	-£178.03
Results section, 225	Upper limit of 95% confidence interval for the above	-£45.00	-£52.02	-£61.16
Results section, 228	LS mean of DLNS sensor layers costs (at 3 months)	£131.00	£151.42	£178.03
Results section, 228	SE of the above	£11.00	£12.71	£14.95
Results section, 228	LS mean of DLNS sensor layers costs (at 6 months)	£115.00	£132.93	£156.29

Results section, 228	SE of the above	£11.00	£12.71	£14.95
Results section, 232	Reduction in LS mean of total ostomy solution costs (at 3 months)	-£4.00	-£4.62	-£5.44
Results section, 232	Lower limit of 95% confidence interval for the above	-£69.00	-£79.76	-£93.77
Results section, 232	Upper limit of 95% confidence interval for the above	£61.00	£70.51	£82.90
Results section, 232	Reduction in LS mean of total ostomy solution costs (at 6 months)	-£2.00	-£2.31	-£2.72
Results section, 232	Lower limit of 95% confidence interval for the above	-£74.00	-£85.54	-£100.57
Results section, 232	Upper limit of 95% confidence interval for the above	£70.00	£80.91	£95.13
Figure 1	Pouching system cost at baseline (no DLNS)	£363.00	£419.59	£493.32

Figure 1	Supporting cost at baseline (no DLNS)	£456.00	£527.09	£619.70
Figure 1	Pouching system cost at 3 months (with DLNS)	£300.00	£346.77	£407.70
Figure 1	Supporting cost at 3 months (with DLNS)	£367.00	£424.22	£498.75
Figure 1	DLNS cost at 3 months	£131.00	£151.42	£178.03
Figure 1	Pouching system cost at 6 months (with DLNS)	£313.00	£361.80	£425.37
Figure 1	Supporting cost at 6 months (with DLNS)	£368.00	£425.37	£500.11
Figure 1	DLNS cost at 6 months	£115.00	£132.93	£156.29
Table 2	LS mean of pouching system costs (baseline)	£363.00	£419.59	£493.32
Table 2	SE of the above	£20.00	£23.12	£27.18
Table 2	LS mean of pouching system costs (at 3 months)	£300.00	£346.77	£407.70
Table 2	SE of the above	£18.00	£20.81	£24.46
Table 2	LS mean of pouching system	£313.00	£361.80	£425.37

	costs (at 6 months)			
Table 2	SE of the above	£20.00	£23.12	£27.18
Table 2	Median of pouching system costs (baseline)	£325.00	£375.67	£441.68
Table 2	Lower quartile limit of the above	£228.00	£263.55	£309.85
Table 2	Upper quartile limit of the above	£460.00	£531.71	£625.14
Table 2	Median of pouching system costs (at 3 months)	£230.00	£265.86	£312.57
Table 2	Lower quartile limit of the above	£188.00	£217.31	£255.49
Table 2	Upper quartile limit of the above	£382.00	£441.55	£519.14
Table 2	Median of pouching system costs (at 6 months)	£260.00	£300.53	£353.34
Table 2	Lower quartile limit of the above	£190.00	£219.62	£258.21
Table 2	Upper quartile limit of the above	£393.00	£454.27	£534.09
Table 2	LS mean difference for pouching system costs (at 3 months)	-£64.00	-£73.98	-£86.98
Table 2	Lower limit of	-£98.00	-£113.28	-£133.18

	95% confidence interval for the above			
Table 2	Upper limit of 95% confidence interval for the above	-£30.00	-£34.68	-£40.77
Table 2	LS mean difference for pouching system costs (at 6 months)	-£50.00	-£57.80	-£67.95
Table 2	Lower limit of 95% confidence interval for the above	-£85.00	-£98.25	-£115.52
Table 2	Upper limit of 95% confidence interval for the above	-£16.00	-£18.49	-£21.74
Table 2	LS mean of supporting product costs (baseline)	£456.00	£527.09	£619.70
Table 2	SE of the above	£29.00	£33.52	£39.41
Table 2	LS mean of supporting products costs (at 3 months)	£367.00	£424.22	£498.75
Table 2	SE of the above	£23.00	£26.59	£31.26
Table 2	LS mean of	£368.00	£425.37	£500.11

	supporting products costs (at 6 months)			
Table 2	SE of the above	£23.00	£26.59	£31.26
Table 2	Median of supporting products costs (baseline)	£394.00	£455.42	£535.45
Table 2	Lower quartile limit of the above	£234.00	£270.48	£318.01
Table 2	Upper quartile limit of the above	£570.00	£658.86	£774.63
Table 2	Median of supporting products costs (at 3 months)	£328.00	£379.14	£445.75
Table 2	Lower quartile limit of the above	£199.00	£230.02	£270.44
Table 2	Upper quartile limit of the above	£450.00	£520.16	£611.55
Table 2	Median of supporting products costs (at 6 months)	£320.00	£369.89	£434.88
Table 2	Lower quartile limit of the above	£229.00	£264.70	£311.21
Table 2	Upper quartile limit of the above	£463.00	£535.18	£629.22
Table 2	LS mean difference for	-£89.00	-£102.88	-£120.95

	supporting products costs (at 3 months)			
Table 2	Lower limit of 95% confidence interval for the above	-£126.00	-£145.64	-£171.23
Table 2	Upper limit of 95% confidence interval for the above	-£52.00	-£60.11	-£70.67
Table 2	LS mean difference for supporting products costs (at 6 months)	-£88.00	-£101.72	-£119.59
Table 2	Lower limit of 95% confidence interval for the above	-£131.00	-£151.42	-£178.03
Table 2	Upper limit of 95% confidence interval for the above	-£45.00	-£52.02	-£61.16
Table 2	LS mean of DLNS sensor layers costs (at 3 months)	£131.00	£151.42	£178.03
Table 2	SE of the above	£11.00	£12.71	£14.95
Table 2	LS mean of DLNS sensor	£115.00	£132.93	£156.29

	layers costs (at 6 months)			
Table 2	SE of the above	£11.00	£12.71	£14.95
Table 2	Median of DLNS sensor layers costs (at 3 months)	£121.00	£139.86	£164.44
Table 2	Lower quartile limit of the above	£53.00	£61.26	£72.03
Table 2	Upper quartile limit of the above	£179.00	£206.91	£243.26
Table 2	Median of DLNS sensor layers costs (at 6 months)	£94.00	£108.65	£127.75
Table 2	Lower quartile limit of the above	£24.00	£27.74	£32.62
Table 2	Upper quartile limit of the above	£181.00	£209.22	£245.98
Table 2	LS mean of total ostomy solution costs (baseline)	£784.00	£906.23	£1,065.46
Table 2	SE of the above	£39.00	£45.08	£53.00
Table 2	LS mean of total ostomy solutions costs (at 3 months)	£779.00	£900.45	£1,058.66
Table 2	SE of the above	£41.00	£47.39	£55.72
Table 2	LS mean of total ostomy	£782.00	£903.91	£1,062.74

	solutions costs (at 6 months)			
Table 2	SE of the above	£43.00	£49.70	£58.44
Table 2	Median of total ostomy solutions costs (baseline)	£799.00	£923.56	£1,085.84
Table 2	Lower quartile limit of the above	£521.00	£602.22	£708.04
Table 2	Upper quartile limit of the above	£1,033.00	£1,194.04	£1,403.85
Table 2	Median of total ostomy solutions costs (at 3 months)	£682.00	£788.32	£926.84
Table 2	Lower quartile limit of the above	£497.00	£574.48	£675.42
Table 2	Upper quartile limit of the above	£1,006.00	£1,162.84	£1,367.15
Table 2	Median of total ostomy solutions costs (at 6 months)	£715.00	£826.47	£971.69
Table 2	Lower quartile limit of the above	£519.00	£599.91	£705.32
Table 2	Upper quartile limit of the above	£1,023.00	£1,182.49	£1,390.26
Table 2	LS mean difference for total ostomy solutions	-£4.00	-£4.62	-£5.44

	costs (at 3 months)			
Table 2	Lower limit of 95% confidence interval for the above	-£60.00	-£69.35	-£81.54
Table 2	Upper limit of 95% confidence interval for the above	£61.00	£70.51	£82.90
Table 2	LS mean difference for total ostomy solutions costs (at 6 months)	-£2.00	-£2.31	-£2.72
Table 2	Lower limit of 95% confidence interval for the above	-£74.00	-£85.54	-£100.57
Table 2	Upper limit of 95% confidence interval for the above	£70.00	£80.91	£95.13
Results section, 244	Decrease from baseline to month 3 in total LS mean costs for stoma-related consultations plus ostomy solution use	-£304.00	-£351.39	-£413.14

Results section, 245	Lower limit of 95% confidence interval for the above	-£456.00	-£527.09	-£619.70
Results section, 245	Upper limit of 95% confidence interval for the above	-£153.00	-£176.85	-£207.93
Results section, 245	Decrease from baseline to month 6 in total LS mean costs for stoma-related consultations plus ostomy solution use	-£340.00	-£393.01	-£462.06
Results section, 245	Lower limit of 95% confidence interval for the above	-£504.00	-£582.57	-£684.94
Results section, 245	Upper limit of 95% confidence interval for the above	-£175.00	-£202.28	-£237.83
Figure 2	LS mean costs for stoma-related consultations plus ostomy solution use at	£1,386.00	£1,602.08	£1,883.57

	baseline (no DLNS)			
Figure 2	LS mean costs for stoma-related consultations plus ostomy solution use at 3 months (with DLNS)	£1,082.00	£1,250.68	£1,470.44
Figure 2	LS mean costs for stoma-related consultations plus ostomy solution use at 6 months (with DLNS)	£1,047.00	£1,210.23	£1,422.87
Figure 2	Decrease from baseline to month 3 in total LS mean costs for stoma-related consultations plus ostomy solution use	-£304.00	-£351.39	-£413.14
Figure 2	Decrease from baseline to month 6 in total LS mean costs for stoma-related consultations plus ostomy	-£340.00	-£393.01	-£462.06

	solution use			
Results section, 255	Result of sensitivity analysis assessing the impact of imputations applied to pouching system and supporting product costs (at baseline)	£4.00	£4.62	£5.44
Results section, 255	Upper limit of sensitivity analysis for the above	£367.00	£424.22	£498.75
Results section, 255	Lower limit of sensitivity analysis for the above	£363.00	£419.59	£493.32
Results section, 255	Result of sensitivity analysis assessing the impact of imputations applied to pouching system and supporting product costs (at 3 months)	£5.00	£5.78	£6.80
Results section, 255	Upper limit of sensitivity analysis for the above	£305.00	£352.55	£414.50

Results section, 255	Lower limit of sensitivity analysis for the above	£300.00	£346.77	£407.70
Results section, 256	Result of sensitivity analysis assessing the impact of imputations applied to pouching system and supporting product costs (at 6 months)	£6.00	£6.94	£8.15
Results section, 256	Upper limit of sensitivity analysis for the above	£319.00	£368.73	£433.52
Results section, 256	Lower limit of sensitivity analysis for the above	£313.00	£361.80	£425.37
Results section, 257	Result of sensitivity analysis assessing the impact of imputations applied to supporting product costs (at baseline)	£269.00	£310.94	£365.57
Results section, 257	Upper limit of sensitivity analysis	£725.00	£838.03	£985.28

	for the above			
Results section, 257	Lower limit of sensitivity analysis for the above	£456.00	£527.09	£619.70
Results section, 257	Result of sensitivity analysis assessing the impact of imputations applied to supporting product costs (at 3 months)	£177.00	£204.59	£240.54
Results section, 257	Upper limit of sensitivity analysis for the above	£544.00	£628.81	£739.30
Results section, 257	Lower limit of sensitivity analysis for the above	£367.00	£424.22	£498.75
Results section, 258	Result of sensitivity analysis assessing the impact of imputations applied to supporting product costs (at 6 months)	£210.00	£242.74	£285.39
Results section, 258	Upper limit of sensitivity analysis for the above	£578.00	£668.11	£785.50

Results section, 258	Lower limit of sensitivity analysis for the above	£368.00	£425.37	£500.11
Results section, 259	Result of sensitivity analysis assessing the impact of imputations applied to total ostomy solution costs (at baseline)	£247.00	£285.51	£335.67
Results section, 259	Upper limit of sensitivity analysis for the above	£1,030.00	£1,190.58	£1,399.77
Results section, 259	Lower limit of sensitivity analysis for the above	£784.00	£906.23	£1,065.46
Results section, 259	Result of sensitivity analysis assessing the impact of imputations applied to total ostomy solution costs (at 3 months)	£176.00	£203.44	£239.18
Results section, 259	Upper limit of sensitivity analysis for the above	£955.00	£1,103.88	£1,297.85

Results section, 259	Lower limit of sensitivity analysis for the above	£779.00	£900.45	£1,058.66
Results section, 259	Result of sensitivity analysis assessing the impact of imputations applied to total ostomy solution costs (at 6 months)	£208.00	£240.43	£282.67
Results section, 259	Upper limit of sensitivity analysis for the above	£989.00	£1,143.19	£1,344.05
Results section, 259	Lower limit of sensitivity analysis for the above	£782.00	£903.91	£1,062.74
Results section, 268	Costs of stoma-related HCP consultations at baseline for newly discharged patients	£679.00	£784.86	£922.76
Results section, 268	Costs of stoma-related HCP consultations at baseline for experienc	£582.00	£672.73	£790.94

	ed patients			
Results section, 270	Costs of stoma-related HCP consultations at 6 months for newly discharged patients	£273.00	£315.56	£371.01
Results section, 270	Costs of stoma-related HCP consultations at 6 months for experienced patients	£215.00	£248.52	£292.19
Results section, 271	Costs of stoma-related hospitalisations at baseline for newly discharged patients	£3,647.00	£4,215.57	£4,956.27
Results section, 272	Costs of stoma-related hospitalisations at baseline for experienced patients	£1,876.00	£2,168.47	£2,549.48
Results section, 272	Costs of stoma-related hospitalisations at 3 months for newly discharged patients	£181.00	£209.22	£245.98

Results section, 272	Costs of stoma-related hospitalisations at 3 months for experienced patients	£1,227.00	£1,418.29	£1,667.49
Results section, 273	Costs of stoma-related hospitalisations at 6 months for newly discharged patients	£436.00	£503.97	£592.52
Results section, 273	Costs of stoma-related hospitalisations at 6 months for experienced patients	£444.00	£513.22	£603.40
Figure 3a	Costs for stoma-related HCP consultations at baseline, all patients	£610.00	£705.10	£828.99
Figure 3a	Costs for stoma-related HCP consultations at baseline, newly discharged patients	£679.00	£784.86	£922.76
Figure 3a	Costs for stoma-related HCP	£582.00	£672.73	£790.94

	consultations at baseline, experienced patients			
Figure 3a	Costs for stoma-related HCP consultations at 3 months, all patients	£301.00	£347.93	£409.06
Figure 3a	Costs for stoma-related HCP consultations at 3 months, newly discharged patients	£300.00	£346.77	£407.70
Figure 3a	Costs for stoma-related HCP consultations at 3 months, experienced patients	£293.00	£338.68	£398.19
Figure 3a	Costs for stoma-related HCP consultations at 6 months, all patients	£258.00	£298.22	£350.62
Figure 3a	Costs for stoma-related HCP consultations at 6 months, newly	£215.00	£248.52	£292.19

	discharged patients			
Figure 3a	Costs for stoma-related HCP consultations at 6 months, experienced patients	£273.00	£315.56	£371.01
Figure 3b	Costs for stoma-related hospitalisations at baseline, all patients	£2,318.00	£2,679.38	£3,150.16
Figure 3b	Costs for stoma-related hospitalisations at baseline, newly discharged patients	£3,647.00	£4,215.57	£4,956.27
Figure 3b	Costs for stoma-related hospitalisations at baseline, experienced patients	£1,876.00	£2,168.47	£2,549.48
Figure 3b	Costs for stoma-related hospitalisations at 3 months, all patients	£877.00	£1,013.72	£1,191.84
Figure 3b	Costs for stoma-related hospitalisations at 3 months,	£181.00	£209.22	£245.98

	newly discharged patients			
Figure 3b	Costs for stoma-related hospitalisations at 3 months, experienced patients	£1,227.00	£1,418.29	£1,667.49
Figure 3b	Costs for stoma-related hospitalisations at 6 months, all patients	£433.00	£500.50	£588.45
Figure 3b	Costs for stoma-related hospitalisations at 6 months, newly discharged patients	£436.00	£503.97	£592.52
Figure 3b	Costs for stoma-related hospitalisations at 6 months, experienced patients	£444.00	£513.22	£603.40
Discussion, 290	Decrease from baseline to month 3 in total LS mean costs for stoma-related HCP consultations	-£309.00	-£357.17	-£419.93
Discussion, 290	Decrease from	-£353.00	-£408.03	-£479.73

	baseline to month 6 in total LS mean costs for stoma-related HCP consultations			
Discussion, 298	Reduction in LS mean of total ostomy solution costs (at 3 months)	-£4.00	-£4.62	-£5.44
Discussion, 298	Reduction in LS mean of total ostomy solution costs (at 6 months)	-£2.00	-£2.31	-£2.72
Discussion, 322	Unadjusted ostomy solution costs at baseline	£1,030.00	£1,190.58	£1,399.77
Discussion, 322	Ostomy solution costs at baseline after ruleset adjustments	£784.00	£906.23	£1,065.46
Discussion, 323	Ostomy solution costs at baseline in the health economic model	£1,547.00	£1,788.18	£2,102.37
			Bank of England exchange rate to EUR on 15th September 2025	Bank of England exchange rate to USD on 15th September 2025

References

1. National Health Service. Drug Tariff. Available online: https://www.nhsbsa.nhs.uk/pharmacies-gp-practices-and-appliance-contractors/drug-tariff (accessed on December 7, 2025).
2. Boisen, E.B.; Cawson, M.; de Fries Jensen, L.; Mealing, S.; van Hest, N. Cost-Effectiveness of a Digital Leakage Notification System (Heylo) for People with Ileostomies or Colostomies in the United Kingdom. Pharmacoeconomics 2025, 43, 955–968, doi:10.1007/s40273-025-01498-9.
3. PrescQIPP. Stoma. Bulletin 338. December 2023. 2.0. Available online: https://www.prescqipp.info/media/2cljvm44/338-stoma-2-0.pdf (accessed on December 4, 2025).
4. National Health Service. 2023/24 National cost collection data publication. Available online: https://www.england.nhs.uk/publication/2023-24-national-cost-collection-data-publication/ (accessed on September 30, 2024).
5. Jones KC, W.H., Birch S, et al. Unit Costs of Health and Social Care 2024 Manual; Kent, United Kingdom; , 2025.
6. National Health Service. Hospital Episode Statistics (HES). Available online: https://digital.nhs.uk/data-and-information/data-tools-and-services/data-services/hospital-episode-statistics (accessed on January 20, 2026).
